# Experiences of Parent Peer Nutrition Educators Sharing Child Feeding and Nutrition Information

**DOI:** 10.3390/children4090078

**Published:** 2017-08-29

**Authors:** Richard Ball, Kerith Duncanson, Tracy Burrows, Clare Collins

**Affiliations:** 1 Mid North Coast Local Health District, Port Macquarie, NSW 2444, Australia; richard.ball@ncahs.health.nsw.gov.au; 2 Department of Nutrition and Dietetics, The University of Newcastle, Newcastle, NSW 2308, Australia; tracy.burrows@newcastle.edu.au; 3 NSW Health Education and Training Institute, NSW Health, Gladesville, NSW 2111, Australia; 4 Priority Research Centre for Physical Activity and Nutrition, The University of Newcastle, Newcastle, NSW 2308, Australia; clare.collins@newcastle.edu.au

**Keywords:** child feeding, peer education, parent, nutrition, social media

## Abstract

The aim of this study was to describe the experiences of parents as peer educators disseminating nutrition and child feeding information. Parents of infants aged from birth to three years were trained as peer educators in a face-to-face workshop, and then shared evidence-based child feeding and nutrition information via Facebook, email, and printed resources for six months to peers, family, and social media contacts. Semi-structured telephone or group interviews were conducted after a six-month online and face-to-face peer nutrition intervention period investigating peer educator experiences, barriers, enablers of information dissemination, and the acceptability of the peer educator model. Transcripts from interviews were independently coded by two researchers and thematically analysed. Twenty-eight participants completed the study and were assigned to either group or individual interviews. The cohort consenting to the study were predominantly female, aged between 25 and 34 years, non-indigenous, tertiary educated, and employed or on maternity leave. Dominant themes to emerge from the interviews included that the information was trustworthy, child feeding practice information was considered most helpful, newer parents were the most receptive and family members the least receptive to child feeding and nutrition information, and sharing and receiving information verbally and via social media were preferred over print and email. In conclusion, parents reported positive experiences as peer nutrition educators, and considered it acceptable for sharing evidence-based nutrition information. Further research may determine the impact on diet quality and the food-related behaviours of babies and young children on a population level.

## 1. Introduction

Dietary patterns, taste preferences, and other food-related behaviours that develop in childhood track into adulthood, contributing to the relative risk of preventable disease [1]. The Australian Health Survey indicates that children aged two to three years consume 35% of the recommended intake of vegetables and consume 30% of their energy (calorie) requirement from energy-dense, nutrient-poor discretionary foods [2]. The same survey indicates that adults meet only 6.8% of the recommended intake for vegetables [2] and consume 35% of energy from discretionary foods. As poor dietary patterns appear to be established before the age of two years [3,4], parents require early and targeted support [5] to get children into healthy eating habits early. Poor child dietary intake is a likely contributor to high and increasing chronic disease, overweight, and obesity rates [1] amongst Australian adults.

Interventions which have reported improvements to children’s eating patterns have been predominantly treatment programs for the families of obese children [1,6,7]. These programs are not appropriate to be delivered at a population level due to the cost, intensity, and different factors influencing the families of healthy weight children [7].

Parents and carers are the biggest influencers on the early life development of children’s eating patterns, taste preferences, and dietary intake [8,9], with the majority of children’s food choices determined by parents or carers until school age [2,10,11]. The food provision practices of parents and carers can either contribute to or hinder children’s development as healthy eaters [9]. Although parents desire good health for their children, they have difficulty translating national dietary guidelines into practice and implementing behaviour management techniques in the current food environment [12].

Parents’ beliefs in relation to child feeding are largely influenced by parenting peer groups [12], with mothers of infants and toddlers citing the internet, family, and friends as their most regular sources of nutrition information [13]. Child feeding practices are therefore more likely to be guided by peer influence and social norms rather than by health professionals [12]. This phenomenon can be explained by the Theory of Planned Behaviour (TPB), which proposes that behaviour is determined by a complex interaction between attitudes, perceived control, normative beliefs, motivation to comply with norms, and interactions between these factors [12]. The TPB has been used in previous qualitative research to assist in understanding child feeding [12].

Peer education has potential to overcome barriers to engaging parents in interventions targeting child and infant nutrition [13,14]. Peer nutrition education has been shown to positively affect the intended target’s behaviours in breast-feeding, weight loss, chronic disease risk, and fruit and vegetable intake [15,16,17,18], although the majority of models have involved education being delivered in a structured setting and requiring a high level of commitment from the peer educator [15,18].

First-time mothers commonly form strong social connections with mothers who have infants of a similar age [8]. Peer groups can produce changes that are more sustained compared to individual changes, as a group’s social support and norms can be more resistant to change [19]. Such groups potentially offer an important system for sharing evidenced-based nutrition information with immediacy, optimal timing, and maximum impact. Peer education may therefore be a useful means of addressing issues of social isolation and delivering a cost-efficient intervention on a population level.

A pilot study of first-time mothers of infants and toddlers conducted by the authors as a precursor to this study indicated a willingness to seek evidence-based nutrition education and undertake two or more hours of training in preparation for on-training their peers [13]. The intention of using a peer educator model in this study was to capitalise on parenting connectedness among peers while maximising flexibility in nutrition education delivery. To the authors’ knowledge, no current studies exist which have investigated parents of infants and toddlers as peer educators, using informal, existing social structures to share nutrition and child feeding information with other parents.

The aims of this study were (1) to describe the experiences of peer educators in delivering nutrition and child feeding information within social networks, (2) to determine the barriers and enablers of peer nutrition education, and (3) to determine peer educators’ perceptions of the acceptability of receiving child nutrition and feeding information from the peer educators.

## 2. Methods

### 2.1. Methodology and Study Design

Qualitative study methods grounded in the Theory of Planned behaviour were used to collect data from peer educators about their experiences and perceptions of peer nutrition education.

### 2.2. Participants and Setting

Participants were recruited from childcare centres and child and family health facilities (March to May 2014). Posters and newsletter advertisements were provided for centres and staff provided information packs to interested parents and carers. Maximum deviation sampling was used to broaden the range of socio-economic and cultural groups represented. Snowball sampling was used to better reach male parents via female partners who had already consented to the study. Eight child care centres catering for an average of 50 families per centre were invited to recruit into the study, and recruitment continued until at least 30 eligible participants had consented.

Inclusion criteria for this study were parents and primary carers:

- With a child aged zero to three years (at study commencement).
- Aged 18 or older.
- Residing within the Mid North Coast Local Health District of New South Wales (NSW), Australia.
- With children whose nutrition/feeding practice requirements are not influenced by a medical condition.
- Ability to understand written and spoken English.

### 2.3. The Intervention

Consenting study participants attended a single two-hour workshop facilitated by the Principal Investigator in one of four locations. The workshop covered the participants’ study role and nutrition and child feeding practices specific to children aged zero to three (Table 1). Following the workshop, the participants explained their study role as peer nutrition educators to friends and family, and shared nutrition and child feeding information throughout the six-month intervention.

The peer educators were provided a folder of printed resources, and a study-specific Facebook [20] page “Food For Kids Mid North Coast” was created to facilitate information sharing. One to four messages were posted per day by the Principal Investigator, totalling 311 posts over the six-month intervention, with additional posts contributed by peer educators or page followers outside the study. The Facebook post content and format was adapted over the six-month intervention period based on ‘post’ performance and feedback. The information and links posted on Facebook were emailed to all peer educators on three occasions staggered evenly throughout the intervention.

The peer educators were contacted by telephone two and four months into the intervention to debrief and to provide feedback on progress and resource needs. They were also encouraged to access the Principal Investigator via telephone or email for additional support.

### 2.4. Procedure

The peer educators provided demographic data at the workshop prior to the intervention and were invited to attend semi-structured group interviews, 60 to 75 min in duration, at the conclusion of the intervention. Those participants who were unable to attend group interviews were offered a telephone interview, 20 to 30 min in duration, as an alternative, after the group interviews had been completed. The group and individual interviews consisted of the same series of questions, focusing on a participant’s experiences as peer educators (Table 2).

The Associate Investigator facilitated all interviews with no one else present aside from the peer educators and the Principal Investigator who took field notes. All interviews were digitally recorded and electronically sent for transcription externally. The peer educators were invited to review transcription contents, but all declined.

### 2.5. Data Analysis

The first transcript was jointly coded for themes by the Principal and Associate Investigators for consistency. The remaining transcripts were systematically coded for themes by the Principal and Associate Investigators independently, and the initial codes, relationships between codes, and emerging themes were then discussed and consolidated into one document for each theme. The codes and associated quotes were then transposed into a spreadsheet revised and refined by the Principal and Associate Investigators until a final consensus on theme and subtheme allocation was reached.

### 2.6. Reflexivity

The Principal Investigator has over 10 years of dietetic experience and extensive experience in children’s nutrition. The Associate Investigator has over 20 years of dietetic experience and extensive knowledge in child feeding and infant nutrition, having completed a Ph.D. in this field of research. Both were known to several participants in both a community and professional context prior to study commencement.

Ethics permission was approved for this study by the North Coast New South Wales Human Research Ethics Committee (No. LNR 084) on 31 March 2014.

## 3. Results

Thirty-four participants consented to peer educator training as part of the study and attended one of four introductory workshops. The retention rate was 82%, as six participants withdrew from the study, leaving 28 participants completing the intervention and participating in group or individual interviews. The cohort consenting to the study were predominantly female, aged between 25 and 34 years, non-indigenous, tertiary educated, and employed or on maternity leave (Table 3).

Thirteen individual interviews were conducted, and two group interviews were attended by nine and six participants.

Four major themes emerged from the group and individual interviews: influences on sharing; sharing mediums; pitching the message; and support for peer educators. Each theme overlapped to some extent with at least one other theme.

### 3.1. Theme 1: Influences on Sharing

‘Influences on sharing’ encompasses factors reported as contributing positively or negatively to the sharing of child feeding and nutrition information with other parents and family.

The stage of parenting (age(s) of parents’ child(ren)) was described by peer educators as a determinant of how receptive parents were to receiving nutrition and child feeding information. First-time parents with very young children were reported as more open and willing to learn, while parents with an older first child or more than one child were considered less receptive.

*“Catching that new parent…is like gold because they are just kind of ‘open’: once you go through a few years of it you feel confident in what you have been doing even if it is not necessarily right”*.

Peer educator, mother of two children, both older than 24 months.

Peer educators reported that their prioritisation of child feeding (and parenting in general) positioned them well to share information with peers and family.

*“We are coming to this because we are probably on the more motivated end of parenting so there are other people that are less educated and have less income and they are less motivated”*.

Peer educator, mother of one 6–8-month-old child and one child older than 24 months.

Peer educators reported increased nutrition knowledge and changes to their own child feeding practices that resulted in positive changes to their own child(ren)’s diet. Such role modelling of child feeding practices was reported to strengthen the impact of child feeding messages compared to sharing messages verbally.

*“Because they have seen me…they are like oh, he is eating more than what he used to and I am like ‘that is because I have stopped hassling him’”*.

Peer educator, mother of one 16–18-month-old child.

Conversely peer educators reported their confidence decreasing if parents expressed strong beliefs that contradicted the evidence-based information being shared.

*“I tended to…be a bit more cautious…I mean my personality is that I tend to not want to get into a conflict or an argument…”*.

Peer educator, mother of one 12–15-month-old child and one child older than 24 months.

Those parents who were perceived as more receptive were more likely to be offered information by peer educators. Receptiveness was considered to be affected by the importance parents placed on child feeding. This was considered challenging if a parent was known to have strong beliefs, attitudes, or opinions about child feeding.

*“The ones who are maybe stuck in their beliefs or believe really heavily that they are doing the right thing …maybe not so much.”*.

Peer educator, mother of one 2–15-month-old child and one child older than 24 months.

Those parents with whom peer educators had closer relationships were reported as easier to share messages with as less familiarity created less certainty of the response.

*“…the first mum's group who I did most of the sharing with, just because I saw them more regularly…We have known each other for a couple of years now”*.

Peer educator, mother of one 0–6-month-old child and one child older than 24 months.

Familiarity appeared to have the opposite effect when family were concerned. The majority of the peer educators reported that family (particularly their parents or partner’s parents) were the most reluctant recipients of information. This caused peer educators much frustration as family were often in caring roles for peer educators’ children.

*“I suspect nothing you could have told me would have helped with my mother....”*.

Peer educator, father of two children older than 24 months.

Peer educators reported a vast array of nutrition and child feeding information available outside of the study, making it difficult to determine which information to trust.

*“They were happy to go along with what their mothers or mother-in-laws were telling them. It did not matter how factual my stuff was”*.

Peer educator, mother of one child aged 6–8 months and three children older than 24 months.

Peer educators who shared nutrition and child feeding information prior to the intervention reported receiving a mixed response. Participation in the study was believed to legitimise information sharing.

*… Some of my friends were… here (she) goes again. But because I had the backing of the access to nutritionists and dietitians, I think they were more happy to listen to me”*.

Peer educator, mother of one 6–8-month-old child and three children older than 24 months.

Lower socio-economic and less-educated parents were perceived as most at need of a child feeding intervention. If the friends and family of peer educators were considered to be at a higher education level, a level of surprise about poor feeding practices was evident.

*“(My) friends are quite well educated but it seems like people are sliding into complacency. If you think about the people at the other end of the spectrum who would not access something like this...”*.

Peer educator, mother of one 16–18-month-old child and three children older than 24 months.

A consistently reported frustration related to the demonstration and justification of poor feeding practices by family, particularly older family members. Older generations generally were considered to have lower standards of what was acceptable practice.

*“One of the mums in my group said ‘…I dished up afternoon snacks of tomatoes and capsicum…and my parents were shocked. They said ‘What are you feeding them? We did not feed you food like that and you guys turned out okay’ and I said ‘Yeah Mum really, well I’m fat’’”*.

Peer educator, mother of one 0–6-month-old child and one child older than 24 months.

Peer educators felt parents would not persist in implementing appropriate child feeding practices due to the extra work required before they were rewarded for effort. When perceived barriers to the difficulty in child feeding were successfully challenged, peer educators reported parents surprise at the simplicity.

*“I think people read it because they are interested in it and then it is like a light bulb moment, like ‘oh, is that all we have to do’”*.

Peer educator, mother of one 16–18-month-old child and three children older than 24 months.

### 3.2. Theme 2: Sharing Mediums

A diverse range of opinions were reported by the peer educators concerning the various sharing mediums (Facebook, email, verbal, and print) used over the intervention.

Verbal information sharing was considered to allow responsive, impromptu information sharing, catering more specifically to parents’ needs than Facebook posts. This was reported by peer educators to be a less overt way to approach sharing information because informal conversations were already happening within groups.

*“The way I approached it was more through my mother's group… just if something happened to come up in conversation I would just go along with it”*.

Peer educator, mother of one 12–15-month-old child.

Peer educators reported that Facebook was an ideal medium for sharing messages with parents who required assistance, without it appearing that they were being targeted. Child feeding and parenting issues in general were described as topics requiring a sensitive approach to avoid distress or conflict.

*“There were a couple of people that I thought of immediately and I tried to tag them in posts that were relevant and share, like just chat about it in general without being too pushy…”*.

Peer educator, mother of one 6–8-month-old child and one child older than 24 months.

Peer educators who were regular Facebook users described the ease with which information could be shared and accessed by parents within parent networks, enabling the optimal timing of information sharing. Facebook use was reported to provide continued engagement in the study and the sharing process. Posts appearing on newsfeeds helped keep nutrition and child feeding at the forefront of peer educators’ consciousness, prompting sharing.

*“It was good … because that would come up on my (Facebook) feed … it was just a neutral way to trigger my thought about food”*.

Peer educator, mother of two children older than 24 months.

The potential for Facebook sharing to reach far beyond the initial target audience of the peer educators and their family and friends was reported by peer educators who described their posts being liked by Facebook friends living overseas. Print and email resources were the least preferred sharing mediums. Email resources were not considered accessible for responsive sharing, while printed resources were reported as cumbersome and redundant.

*“The actual written information, I did not really use that a lot just because of being time-poor and I guess never getting round to it”*.

Peer educator, mother of one 6–8-month-old child and one child older than 24 months.

### 3.3. Theme 3: The Message and the Pitch

The information and resources provided at the introductory workshop and via Facebook and email focused equally on children’s nutrition and child feeding practices.

Peer educators reported that the focus of interest from themselves and parents was on the child feeding practices. These were reported to provide parents with new, simple, and effective feeding strategies to implement. The positive response from parents about the child feeding information motivated the peer educators to share additional child feeding information. In contrast, it was felt that the nutrition messages were very familiar but hard to implement without accompanying behavioural strategies.

*“‘This is good…that is not’. People have heard that all before. I found the biggest thing for me that was new and made people sit up and listen was the child feeding practices”*.

Peer educator, mother of 16–18-month-old twins and two children older than 24 months.

Educators identified a range of factors relating to message presentation and ‘marketing’, which determined whether posts would attract attention. Parents were described as being time- and energy-poor and messages requiring minimal effort to engage with were considered most popular.

*“The ones that were quite short and straight to the point I shared and found that a lot of the people in my social group had actually seen them…”*.

Peer educator, mother of one 19–21-month-old child and one child older than 24 months.

Messages emphasising parent benefits in addition to child benefits were considered easier to share. In particular, the ‘division of responsibility’ message, emphasising children taking responsibility for how much they eat, was seen to reduce parents’ tension related to feeding.

*“We are selfish creatures are not we? …what makes it easier for me as a parent”*.

Peer educator, mother of one 6–8-month-old child and one child older than 24 months.

### 3.4. Theme 4: Level and Nature of Support for Peer Educators

Feeling supported was reported to be provided from a variety of sources and was a determining factor in whether the research experience was positive or negative for the peer educators.

Interest and positive early interactions from other parents and family was reported to act as a catalyst to progress child feeding conversation, creating a snowball effect.

*“Members of playgroup were always very receptive and everyone was on very similar pages…Information sharing was two-way…”*.

Peer educator, mother of one 9–11-month-old child.

Positive feedback from parents after implementing suggested feeding strategies strengthened peer educators resolve and was described as providing additional support and meaning to involvement in the study. Several peer educators reported that joining the study with a friend or their partner provided them with additional momentum and less pressure to find support within peer groups than others participating alone.

*“A couple of the mums went to the workshop…so having those numbers of people, a certain number… a critical mass”*.

Peer educator, mother of one 6–8-month-old child and one child older than 24 months.

Peer educators reported feeling adequately supported by the research team and being part of the study and were confident in offering to source additional information from the Principal Investigator.

*“I did often say to them ’look if you are unsure or cannot find what you are looking for, just let me know, I can pass it on’”*.

Peer educator, mother of one 9–11-month-old child.

## 4. Discussion

This study strongly supports the feasibility of peer education as a means of disseminating nutrition and child feeding information between the parents of children aged up to three years. Peer educators’ positive experiences and the high retention rate of 82% were indicative of strong engagement, and compares favourably with previous peer educator nutrition programs [18,22,23,24].

### 4.1. The Model

The peer nutrition education model utilised the social structures and communication mediums already in use by parents. The flexibility in the nature and content of the resource materials and mediums were reported by peer educators to be enablers in the dissemination of relevant and applicable information topics [15,17,24]. Previously described peer nutrition education models, in which information is delivered in more formal settings, such as groups, classes, and home visits, are likely to have been less feasible for the peer educators and the parents they were engaging with.

The intervention was much less resource intensive than previously described peer educator models [15,18,22]. Peer educator contact time was intentionally limited to one two-hour workshop and two follow-up calls, representative of the estimated time commitment parents were willing and expected to contribute [10]. This appeared to be compensated for by the perception of support reported by peer educators, possibly due to daily prompts by way of Facebook posts.

This model of intervention proved to be mutually beneficial for the researchers, peer educators, and parents. Those peer educators passionate about nutrition and their children’s health were able to pass on credible evidence-based information, allowing the research team to infiltrate parents’ inner circle, an area difficult to access with correct information [10].

### 4.2. The Mediums

The “Food For Kids Mid North Coast” Facebook page was created specifically for peer educators in this study to share information with their friends and family. However, the distribution of information was reported as being much wider than the target audience, which differs from other educator models [15,18,22]. Peer educators who were using Facebook to discretely share messages considered uncomfortable to discuss face-to-face is consistent with previously reported advantages of online information sharing [25]. However, messages via Facebook were considered less likely to be received and considered by the intended target parents when compared to more direct or tailored approaches.

This provides support for the concept of multi-modal dissemination.

Those peer educators who did not access Facebook may have received an insufficient dose of intervention, particularly if they did not access the print resources or emails. An ample dosage of the message has previously been reported to be an enabler for change [26].

Sharing information verbally allowed peer educators to fully capitalise on their peer relationship advantage, as information could be tailored, taking into consideration the recipient’s needs [26]. Parents have been found to be more likely to change their child feeding-related behaviours if educators share similar demographics and similar nutritional concerns [8]. The strong preference reported by peer educators for sharing messages via Facebook and verbally rather than by print is consistent with literature outlining the limited value parents place in print resources alone [27]. The proliferation of smart phones and the internet has dramatically increased the convenience of accessing health information [25], an important consideration when trying to access time-poor new parents.

### 4.3. The Messages

Trust in known and respected social media sites and content is well-documented to be an enabler for their effective application as a health information source [13,28]. Peer educators reported that the Facebook page created achieved this in an environment where health information sites compete for credibility with non-evidence-based alternative information.

While consistent with previous studies [12,29], the strength of the findings about the reported popularity of child feeding practices information amongst peer educators, parents, and family was surprising. Duncanson et al. found that parents intend to feed their children well but are often unable to convert their intention into practice [12,29].

It is understandable that the child feeding practices information was well-received, as it offered parents alternative feeding strategies when others had been exhausted and the prospect of parents benefitting as well as children. This finding provides an opportunity for health promotion services to expand or redirect the focus of nutrition interventions from purely nutrition and dietary intake towards child feeding practices.

### 4.4. The People

Difficulties reaching vulnerable groups have been reported in other peer educator projects [22], and despite the use of maximum deviation sampling to reach these groups, the study cohort was predominantly female, non-indigenous, and tertiary educated. Peer educators were surprised that inappropriate feeding practices and child dietary intake were not necessarily related to socio-economic status. This differs from previous research which identifies socio-economic factors, such as education level, as predictive of child dietary intake [30].

A fear of being labelled a ‘bad parent’ has been identified to be a barrier to parents engaging in behavioural change [31]. This may explain a reluctance to engage in the sharing process by some parents of older children, as acknowledging a need for information may be perceived as an admission of parenting inadequacy [32]. Alternately, parents may have entrenched ideas about barriers to improving child feeding and dietary intake that become progressively harder to change. Parent groups comprising newer parents or younger children involved more discussion about feeding and parenting in general. New parents may be more motivated to learn appropriate feeding or less guarded and more willing to reveal themselves as novices in both feeding and parenting.

Feeling supported had a direct effect on peer educators’ confidence to share information. The “critical mass” of support from peer educators, family, partners, and other parents was described as a powerful enabler. Parents were able to show their support by engaging and adopting a peer educator’s recommendations. Teamwork and mentoring amongst peer educators has been identified by Hibbs and Sandmann [33] to be a key area to strengthen programs.

The information-sharing dynamic between peer educators and parents depended on the child feeding confidence and efficacy of the peer educators relative to the attitudes and beliefs of parents. The type of responses peer educators received from sharing information with parents appeared to be dealt with differently depending on the relationship type. If shared information was received negatively by parents it was reported more likely to affect ongoing sharing than if the negative response was from family. The risk of negatively impacting on a peer relationship was generally considered too great, whereas negative responses from family were more likely to be accepted and challenged by peer educators.

Participants’ perceptions of the inappropriate child feeding practices of extended family and in particular grandparents are concerning, as they can have a strong influence on the dietary intake of infants and young children [34]. Grandparents play a significant direct caring role for 30% of Australian children with two working parents [35]. Ways to address the inappropriate child feeding practices of some grandparents were not identified in this study and need to be explored further. Peer education of grandparents by non-family members or peer nutrition educator programs specifically for grandparents are recommended areas for future interventions.

## 5. Strengths

To the authors’ knowledge, this is the first study that targets new parents and the development of eating patterns while capitalising on the social structures existing in this demographic group. This study demonstrates the potential of a resource-efficient, evidence-based program to fill a gap in service provision, which extends outside existing service delivery models. The researchers’ professional background, life stage, and immersion in the studied community allowed strong relationship development, insight, and understanding of the issues within the social context of new parents.

## 6. Limitations

The study findings are limited by the demographic of the study peer educators being predominantly rurally located and tertiary educated females. It is possible that the familiarity of the researchers with the research cohort and presence at the group interviews may have impacted some responses, although peer educators were adamant that this was not the case. The inconsistency of the data collection process, with parents completing either group or individual interviews, was a study limitation. This method was used to maximise participation in interviews in consideration of parents’ limited time availability. A comparison of the responses between the group and individual interviews did not reveal any discrepancies or inconsistencies in the responses.

## 7. Implications for Research and Practice

Peer education with a social media component provides an avenue to distribute evidence-based nutrition and child feeding information more widely than conventional interventions with less investment of resources. The high retention rate (82%) of this study demonstrated the acceptability of a flexible model and a minimal time commitment. This outcome suggests that it is possible to engage and retain peer educators if they feel adequately supported, even if the input from researchers is not high.

Facebook was established as an effective and trusted medium for health professionals to share information with parents. This preference for social media over print and email emphasised a need to reconsider traditional ways of communicating health messages and for health services to consider their social media policies. A more strategic and sophisticated social media strategy for peer education models, involving a wider range of platforms, should be employed to further increase reach.

The popularity of the child feeding practices was widely reported by parents and peer educators. The unfamiliarity of parents with recommended feeding practices and the important role they play in assisting feeding intention highlights a need for child feeding practices to be the focus of future programs.

This study identified the requirement for interventions to target parents earlier within the parenting cycle, before feeding practices have been consolidated. Future peer education models should to be run in partnership with child and family health teams who have access to parents at an early stage of the parenting cycle, or even in the antenatal setting. Extension of the study would allow for the evaluation of the reach and scope of the model over an extended intervention period and geographic area, allowing additional time for sustainable changes in child feeding and parenting approaches. Additional recruitment strategies are required to engage marginalised parents. However, the study findings do indicate that a peer education model is more likely to reach marginalised parents in a non-threatening way through secondary and tertiary sharing than existing strategies targeting these groups directly.

Family, and in particular older family, were identified as having an important role in influencing the dietary patterns of young children. Further research is required to investigate how to effectively target extended family in order for them to positively influence feeding practices.

Further investigation should be conducted into the perspective of the parents receiving information from peer educators. Measurement of the effect of a recipient’s behavioural intention, actual behaviour, and changes to their children’s diet quality would make a valuable contribution to this field of research. A cost effectiveness analysis would provide valuable additional and novel information to this field of research.

The results of this study indicate that it is feasible for a peer nutrition education model to be used to upskill parents as peer educators, and that further exploration is required to determine whether the broader implementation of the model can influence the diet quality and food behaviour of babies and young children. To address the impending chronic disease consequences of poor child nutrition, an investment in the development of this resource-efficient peer nutrition education model is warranted.

## Figures and Tables

**Table 1:** Peer nutrition education workshop content and resources for the “Food for Kids Mid North Coast” study.

Peer nutrition educator workshop content	Project background/context
	Theory behind study
	Project timeline
	Project boundaries
	Referral pathways/dietitian contact details
	Complaint procedure
	Children’s health nutrition intake data
	Food environment challenges
	Key contact details
	Sharing information
	Mediums to be used
	Risk management
Peer nutrition educator workshop, print resource nutrition and child feeding education content	Children’s health nutrition intake data
	Food environment challenges
	Evidence and non-evidence based science
	Starting solids
	Australian Guide to Healthy Eating for Children [21]
	Core and non-core foods
	Child feeding practices
	Responsibility—My responsibilities card (division of responsibility)
	Monitoring
	Restriction
	Rewarding
	Environment
	Pressure to eat
	Role modelling
	Exposure
	Defining fussy, picky and problem eating
	Managing food refusal, managing faddy eating
	Recipes/food ideas
	Food safety
	Understanding food labels
	Useful websites list

**Table 2:** “Food For Kid Mid North Coast” study post-intervention group and individual participant interview questions.

Appropriateness	• How well (or not) was the information provided pitched for your friends or family?
	• Can you think of any examples of topics or ideas that were more or less suitable/easy to share?
	• How do you think the information was received by your peers?
	• Were there any factors that made sharing information easier or harder?
Attitudes and beliefs	• What were the attitudes of other parents towards receiving nutrition education?
	• Were there any strong dietary beliefs exhibited by parents or family?
Outcomes/results	• Please comment on the impact the information you shared with parents had on themselves or their children.
	• Please comment on any changes you noticed as a result of the information you shared or due to you being a part of the project.
Logistics	• Please comment on the six-month timeframe of the intervention.
	• What was the timing of this project like in relation to age of your child?

**Table 3:** Demographic characteristics of participants in the “Food For Kids Mid North Coast study” (*n* = 34).

**Parent Gender**			**Parent Age Range**			**Indigenous Status**					
Male	4	(12%)	25–34 years	25	(74%)	Indigenous	1	(3%)			
Female	30	(88%)	35–44 years	9	(26%)	Non-indigenous	33	(97%)			
**Parent Education**			**Employment Status**			**Number of Children**			**Age—Youngest Child**		
University	22	(65%)	Full time	7	(21%)	One child	11	(32%)	0–8 months	11	(32%)
Trade/vocational	5	(15%)	Part time	14	(41%)	Two children	18	(52%)	9–15 months	8	(24%)
Year 12	5	(15%)	Maternity leave	8	(24%)	Three children	3	(8%)	16–23 months	8	(24%)
Other	2	(5%)	Not working	5	(15%)	Four Children	2	(5%)	Over 24 months	6	(18%)

## Supplementary Materials

Supplementary File 1

## References

[1] Albright A. (2008). Biological and social exposures in youth set the stage for premature chronic disease. J. Am. Diet. Assoc..

[2] ABS (2014). Australian Bureau of Statistics, Australian Health Survey: Nutrition First Results—Foods and Nutrients, 2011–12.

[3] Devaney B., Ziegler P., Pac S., Karwe V., Barr S.I. (2004). Nutrient intakes of infants and toddlers. J. Am. Diet. Assoc..

[4] Webb K.L., Lahti-Koski M., Rutishauser I., Hector D.J., Knezevic N., Gill T., Peat J.K., Leeder S.R. (2006). Consumption of ‘extra’ foods (energy-dense, nutrient-poor) among children aged 16–24 months from Western Sydney, Australia. Public Health Nutr..

[5] Burrows T., Hutchesson M., Chai L.K., Rollo M., Skinner G., Collins C. (2015). Nutrition Interventions for Prevention and Management of Childhood Obesity: What Do Parents Want from an eHealth Program?. Nutrients.

[6] Collins C.E., Warren J., Neve M., McCoy P., Stokes B.J. (2006). Measuring effectiveness of dietetic interventions in child obesity: A systematic review of randomized trials. Arch. Pediatrics Adolesc. Med..

[7] Ho M., Garnett S.P., Baur L.A., Burrows T., Stewart L., Neve M., Collins C. (2013). Impact of dietary and exercise interventions on weight change and metabolic outcomes in obese children and adolescents: A systematic review and meta-analysis of randomized trials. JAMA Pediatr..

[8] Cameron A.J., Hesketh K., Ball K., Crawford D., Campbell K.J. (2010). Influence of Peers on Breastfeeding Discontinuation Among New Parents: The Melbourne InFANT Program. Pediatrics.

[9] Wolfenden L., Wyse R.J., Britton B.I., Campbell K.J., Hodder R.K., Stacey F.G., McElduff P., James E.L. (2012). Interventions for increasing fruit and vegetable consumption in children aged 5 years and under. Cochrane Database Syst. Rev..

[10] Ho M., Garnett S.P., Baur L., Burrows T., Stewart L., Neve M., Collins C. (2012). Effectiveness of lifestyle interventions in child obesity: Systematic review with meta-analysis. Pediatrics.

[11] Huybrechts I., Matthys C., Vereecken C., Maes L., Temme E.H., Van Oyen H., De Backer G., De Henauw S. (2008). Food Intakes by Preschool Children in Flanders Compared with Dietary Guidelines. Int. J. Environ. Res. Public Health Nutr..

[12] Duncanson K., Burrows T., Holman B., Collins C. (2013). Parents’ Perceptions of Child Feeding: A Qualitative Study Based on the Theory of Planned Behavior. J. Dev. Behav. Pediatr..

[13] Duncanson K., Burrows T., Collins C. (2014). Peer education is a feasible method of disseminating information related to child nutrition and feeding between new mothers. BMC Public Health.

[14] Cann W., Parenting Research Centre (2009). Raising Children.

[15] Goldfinger J.Z., Arniella G., Wylie-Rosett J., Horowitz C.R. (2008). Project HEAL: Peer Education Leads to Weight Loss in Harlem. J. Health Care Poor Underserved.

[16] Morton D., Rankin P., Morey P., Kent L., Hurlow T., Chang E., Diehl H.A. (2013). The effectiveness of the Complete Health Improvement Program (CHIP) in Australasia for reducing selected chronic disease risk factors: A feasibility study. N. Z. Med. J..

[17] Rempel L., Moore K. (2012). Peer-led prenatal breast-feeding education: A viable alternative to nurse-led education. Midwifery.

[18] White S., Park Y.S., Israel T., Cordero E.D. (2009). Longitudinal evaluation of peer health education on a college campus: Impact on health behaviors. J. Am. Coll. Health.

[19] Buller D.B., Morrill C., Taren D., Aickin M., Sennott-Miller L., Buller M.K., Larkey L., Alatorre C., Wentzel T.M. (1999). Randomized Trial Testing the Effect of Peer Education at Increasing Fruit and Vegetable Intake. J. Natl. Cancer Inst..

[20] Facebook. https://www.facebook.com/.

[21] National Health and Medical Research Council (2013). Australian Guide to Healthy Eating.

[22] Gibson S. (2007). Food Standards Agency peer-led approaches to dietary change. Public Health Nutr..

[23] Mellanby A.R., Newcombe R.G., Rees J., Tripp J.H. (2001). A comparative study of peer-led and adult-led school sex education. Health Educ. Res..

[24] Taylor T., Serrano E., Anderson J. (2001). Management issues related to effectively implementing a nutrition education program using peer educators. J. Nutr. Educ..

[25] Leak T.M., Benavente L., Goodell L.S., Lassiter A., Jones L., Bowen S. (2014). EFNEP Graduates’ perspectives on social media to supplement nutrition education: Focus group findings from active users. J. Nutr. Educ. Behav..

[26] Jepson R., Clegg A., Forbes C., Lewis R., Sowden A., Kleijnen J. (2000). The determinants of screening uptake and interventions for increasing uptake: A systematic review. Health Technol. Assess..

[27] Kunkel M.E., Bell L.B., Luccia B.H. (2001). Peer nutrition education program to improve nutrition knowledge of female collegiate athletes. J. Nutr. Educ..

[28] Tobey L., Manore M. (2013). Social media and nutrition education: The Food Hero experience. J. Nutr. Educ. Behav..

[29] O’Brien M. (1996). Child-rearing difficulties reported by parents of Infants and Toddlers. J. Pediatr. Psychol..

[30] Darmon N., Drewnowski A. (2008). Does social class predict diet quality?. Am. J. Clin. Nutr..

[31] Parenting subgroup, NSW Health (2011). NSW Population Health Healthy Weight Network, Guiding Principles for Engaging and Communicating with Parents and Families in Promotion of Healthy Weight.

[32] O’Key V., Hugh-Jones S. (2010). I don’t need anybody to tell me what I should be doing’. A discursive analysis of maternal accounts of (mis)trust of healthy eating information. Appetite.

[33] Hibbs J., Sandmann L. (2011). Psychosocial impact of training and work experience on EFNEP paraprofessionals. J. Ext..

[34] Roberts M., Pettigrew S. (2010). The influence of grandparents on children’s diets. J. Res. Consum..

[35] Australian Bureau of Statistics. http://www.abs.gov.au/ausstats/abs@.nsf/mediareleasesbytitle/B80CB3BDAC6944AECA257601001B62F7?OpenDocument.

