# Supplementary material for: Experiences of Parent Peer Nutrition Educators Sharing Child Feeding and Nutrition Information
Source: Children (Basel). 2017 Aug 29;4(9):78. doi: 10.3390/children4090078 (PMC5615268; doi:10.3390/children4090078)
Supplement: Supplementary File 1 [file children-04-00078-s001.zip › Table 3 .docx]

**Table 3: Demographic characteristics of participants in the “Food For Kids Mid North Coast study” (n = 34)**

| **Parent gender** |  |  | **Parent age range** |  |  | **Indigenous status** |  |  |  |  |  |
| --- | --- | --- | --- | --- | --- | --- | --- | --- | --- | --- | --- |
| Male | 4 | (12%) | 25-34 years | 25 | (74%) | Indigenous | 1 | (3%) |  |  |  |
| Female | 30 | (88%) | 35-44 | 9 | (26%) | Non - Indigenous | 33 | (97%) |  |  |  |
| **Parent education** |  |  | **Employment status** |  |  | **Number of children** |  |  | **Age - youngest child** |  |  |
| University | 22 | (65%) | Full time | 7 | (21%) | One child | 11 | (32%) | 0-8 months | 11 | (32%) |
| Trade/vocational | 5 | (15%) | Part time | 14 | (41%) | Two children | 18 | (52%) | 9-15 months | 8 | (24%) |
| Year 12 | 5 | (15%) | Maternity leave | 8 | (24%) | Three children | 3 | (8%) | 16-23 months | 8 | (24%) |
| Other | 2 | (5%) | Not working | 5 | (15%) | Four Children | 2 | (5%) | Over 24 months | 6 | (18%) |
|  |  |  |  |  |  |  |  |  |  |  |  |
