# Supplementary material for: Experiences of Parent Peer Nutrition Educators Sharing Child Feeding and Nutrition Information
Source: Children (Basel). 2017 Aug 29;4(9):78. doi: 10.3390/children4090078 (PMC5615268; doi:10.3390/children4090078)
Supplement: Supplementary File 1 [file children-04-00078-s001.zip › Non Blinded Title Page.docx]

Unblinded Title Page

Title: Experiences of parent peer nutrition educators sharing child feeding and nutrition information

Running Head: Peer educator experiences nutrition and child feeding

Corresponding author:

Richard Ball MND (Master of Nutrition and Dietetics)

Port Macquarie Community Health Centre

PO Box 126 Port Macquarie NSW 2444 Australia

Ph. 61 2 6588 2933 Fax 61 2 6588 2837

Richard.ball@ncahs.health.nsw.gov.au

Kerith Duncanson PhD

Department of Nutrition and Dietetics, The University of Newcastle, Newcastle, NSW

NSW Health Education and Training Institute, NSW Health, NSW

Tracy Burrows PhD,

Department of Nutrition and Dietetics, The University of Newcastle, Newcastle, NSW

Clare Collins, PhD

Department of Nutrition and Dietetics, The University of Newcastle, Newcastle, NSW

The authors have been supported conduct this research by the NSW Health Education and Training Institute Rural and Remote Portfolio and the Mid North Coast Local Health District of NSW Health.

The authors have no conflicts of interest to declare

Acknowledgments

The authors would like to thank the participants, for their generosity in sharing their time and experiences. They also like to thank Emma Webster, David Schmidt, from the Health Education and Training Institute.
