# Supplementary material for: Experiences of Parent Peer Nutrition Educators Sharing Child Feeding and Nutrition Information
Source: Children (Basel). 2017 Aug 29;4(9):78. doi: 10.3390/children4090078 (PMC5615268; doi:10.3390/children4090078)
Supplement: Supplementary File 1 [file children-04-00078-s001.zip › Table 1 .docx]

Table 1: Peer nutrition education workshop content and resources for the “Food for Kids Mid North Coast” study

| Peer Nutrition Educator workshop content | Project background/context  Theory behind study  Project time line  Project boundaries  Referral pathways/Dietitian contact details  Complaint procedure  Children’s health nutrition intake data  Food environment challenges  Key contact details  Sharing Information  Mediums to be used  Risk management |
| --- | --- |
| Peer Nutrition Educator workshop, Print Resource Nutrition and child feeding education content | Children’s health nutrition intake data  Food environment challenges  Evidence and non – evidence based science  Starting solids  Australian Guide to Healthy Eating for Children  Core – Non-core foods  Child feeding practices  Responsibility - My responsibilities card (division of responsibility)  Monitoring  Restriction  Rewarding  Environment  Pressure to eat  Role Modelling  Exposure  Picky v problem eaters  Managing food refusal, Managing faddy eating  Recipes/Food ideas  Food safety  Understanding food labels  Useful websites list |
