# Supplementary material for: Experiences of Parent Peer Nutrition Educators Sharing Child Feeding and Nutrition Information
Source: Children (Basel). 2017 Aug 29;4(9):78. doi: 10.3390/children4090078 (PMC5615268; doi:10.3390/children4090078)
Supplement: Supplementary File 1 [file children-04-00078-s001.zip › Table 2 .docx]

**Table 2: “Food For Kid Mid North Coast” study post-intervention group and individual participant interview questions**

| Appropriateness | - How well (or not) was the information provided pitched for your friends or family? - Can you think of any examples of topics or ideas that were more or less suitable/easy to share? - How do you think the information was received by your peers? - Were there any factors that made sharing information easier or harder? |
| --- | --- |
| Attitudes and beliefs | - What were the attitudes of other parents towards receiving nutrition education? - Were there any strong dietary beliefs exhibited by parents or family? |
| Outcomes / results | - Please comment on the impact the information you shared with parents had on themselves or their children. - Please comment on any changes you noticed as a result of information you shared or due to you being a part of the project? |
| logistics | - Please comment on the 6 month timeframe of the intervention. - What was timing of this project like in relation to age of your child? |
